# Significance of the Hematological Scoring System (HSS) in the Early Diagnosis of Neonatal Sepsis in a Tertiary Care Center

**DOI:** 10.7759/cureus.86014

**Published:** 2025-06-14

**Authors:** Rajendra Kushwaha, Lal Pranay Singh, Jyoti Yadav, Deepshikha Verma

**Affiliations:** 1 Department of Pathology, Gandhi Medical College, Bhopal, IND; 2 Department of Pathology, Atal Bihari Vajpayee Government Medical College, Vidisha, IND

**Keywords:** blood culture, early diagnosis of sepsis, hematological scoring system, hematology, neonatal sepsis, peripheral smear, polymorphonuclear count

## Abstract

Background: Most of the neonatal mortality in developing nations is due to sepsis, making it the main cause of death in this age group. The generic characteristics of the clinical symptoms of newborn sepsis make early detection difficult. Blood culture is the gold standard for diagnosis, but it can be expensive and time-consuming, lasting several days. Another technique for the early detection of neonatal sepsis is the Hematological Scoring System (HSS), which utilizes hematologic characteristics such as leukocyte count, polymorphonuclear neutrophil (PMN) cells, immature PMN count, degenerative changes, and platelet count. The purpose of this study was to ascertain the importance of the HSS in detecting newborn sepsis early.

Materials and methods: The present study was a prospective observational study involving infants (>72 hours to 28 days of age) admitted to the Neonatal Intensive Care Unit of Gandhi Medical College and Associated Hospitals, Bhopal. This study was done in the Department of Pathology from January 2020 to June 2021. The total sample size was 153 neonates who were clinically suspected of sepsis. Hematological parameters were measured for all subjects, and each case was analyzed using the HSS.

Results: There were 112 (73.2%) males and 39 (26.8%) females in the clinically diagnosed cases of late-onset sepsis. A total of 75 (49%) of the clinically confirmed cases of septicemia had positive blood cultures. When it came to detecting newborns with sepsis, the total PMN count (74.70%) was the most sensitive, followed by the immature PMN count (65.33%). Platelet counts (61.54%) and the total leukocyte count (TLC) (92%) were highly specific assays that were useful in the diagnosis of sepsis. The immature-to-total PMN ratio (71%) came next. In order to identify infants who actually had sepsis, the total PMN ratio (89.87%) and platelet count (53%) had a significant positive predictive value for immaturity.

Conclusions: HSS is an extremely sensitive indication of sepsis. Its usage in peripheral smear tests can be a useful sepsis screening tool for early diagnosis, lowering newborn morbidity and mortality.

## Introduction

Sepsis is a severe concern in newborns. The vague clinical presentation of newborn septicemia makes early diagnosis challenging. Neonatal sepsis is the most common cause of neonatal illness and mortality in underdeveloped nations like India [1,2]. Neonatal sepsis requires prompt identification because the infection progresses more quickly than in adults. Neonatal sepsis is more prevalent in underdeveloped nations due to risk factors such as low birth weight, prematurity, low Apgar (appearance, pulse, grimace, activity, and respiration) scores at birth, and low socioeconomic status. Other risk factors include premature rupture of membranes (PROM), chorioamnionitis or endometritis, group B Streptococcal colonization, foul-smelling liquor, and meconium-stained amniotic fluid [3]. In poor countries, the mortality rate ranges from 10 to 70 per 1000 live births. If diagnosed promptly and managed with appropriate antimicrobial therapy, neonatal sepsis is a treatable condition with favorable outcomes. However, early detection of sepsis remains a significant difficulty. Because of the inability to diagnose sepsis earlier, antibiotics are used unnecessarily and for an extended period of time. This increases the risk of antibiotic-associated adverse effects and contributes to the emergence of antimicrobial resistance.

Newborns have a weakened immune system, and they are more vulnerable to invasive infections. Infections are more common in premature babies than in full-term babies [4]. Blood culture, haptoglobin, and immune-electrophoresis are costly and time-consuming tests that have a high positive predictive value for detecting sepsis [5]. A positive blood culture provides a clear and confirmed diagnosis, although it is a time-consuming test. Early sepsis diagnosis is achieved by using cytokines, acute-phase proteins, cell surface antigens, and bacterial genomes. Despite their sensitivity and specificity, these markers are costly and hard to find in environments with limited resources. As a result, optimal diagnostic tests should provide speedy results and have high sensitivity and specificity to avoid the use of unnecessary antibiotics [6,7].

The purpose of this study was to determine the diagnostic effectiveness of the Hematological Scoring System (HSS) as a cost-effective screening tool and to determine the significance of the system for early diagnosis of late-onset newborn sepsis.

## Materials and methods

The present study was a prospective observational study involving infants (>72 hours to 28 days of age) admitted to the Neonatal Intensive Care Unit of Gandhi Medical College and Associated Hospitals, Bhopal. This study was done in the Department of Pathology from January 2020 to June 2021. The total sample size was 153 cases.

A power analysis was conducted using the chi-square goodness-of-fit test to determine whether the sample size of 153 neonates was adequate for detecting associations between HSS scores and sepsis status. Assuming a moderate effect size (Cohen’s w = 0.3), a significance level (α) of 0.05, and a sample size of 153, the calculated statistical power was approximately 92%, which exceeds the conventional 80% threshold for adequacy. This confirms that the study was sufficiently powered to detect statistically significant relationships between hematological parameters and neonatal sepsis.

Newborns aged >72 hours to 28 days, as well as those with clinical symptoms and signs of septicemia, were eligible to participate in the study. Exclusion criteria included significant congenital anomalies, inborn metabolic abnormalities, neonates who had received antibiotics, and neonates who had a blood transfusion. The blood samples, collected by peripheral venipuncture using aseptic precautions before starting antibiotics, were sent to the pathology laboratory in ethylenediaminetetraacetic acid (EDTA) vacutainers. Blood smear was prepared within one to two hours of venipuncture. The Leishman stain was used to stain the air-dried smear in our study.

The Mindray (BC5300/BC3600) Hematology Analyzer (Mindray, Gurugram, India) was used to calculate the total white blood cell (WBC) count and platelet count. The total WBC count for nucleated red blood cells was adjusted. Manual checks were performed on the differential count and neutrophilic changes. The total leukocyte count (TLC), total polymorphonuclear neutrophil (PMN) count, immature polymorphonuclear neutrophil (iPMN) count, immature-to-total (I:T) PMN ratio, immature-to-mature (I:M) PMN ratio, platelet count, and degenerative or toxic changes in neutrophils were all assessed and scored.

The following seven parameters received a score of 1 if any of them were abnormal: platelet count (<150,000/cumm), raised immature PMN count (>600), high immature-to-total (I:T) PMN ratio (>0.12), elevated immature-to-mature (I:M) PMN ratio (>0.3), abnormal TLC, abnormal total PMN, and degenerative alterations seen in neutrophils, including toxic granulations, vacuolations, and Döhle bodies.

To compensate for the low I:M ratio, an abnormal total PMN count was awarded a score of 2 if there were no mature neutrophils. The reference values of the hematological parameters of Rodwell et al. were used as the standard values, as shown in Table 1 [8].

**Table 1:** Modified hematological scoring criteria for neonatal sepsis. The scoring system was adapted from Rodwell et al. (1988) [8] and modified according to our study.

Parameter	Abnormal finding	Score
Total white blood cell (WBC) count	<5000/mm³ or elevated age-specific counts: – >25,000 (at birth) – >30,000 (12–24 hours) – >21,000 (after 2 days)	1
Total polymorphonuclear neutrophil (PMN) count	Increased or decreased from the normal range. If no mature PMNs are observed, assign a score of 2	1–2
Immature polymorphonuclear neutrophil (iPMN) count	Elevated immature neutrophils	1
Immature-to-total neutrophil ratio (I:T)	Greater than 0.12	1
Immature-to-mature neutrophil ratio (I:M)	Greater than 0.30	1
Degenerative neutrophil changes	Presence of toxic granulation, cytoplasmic vacuolization, or Döhle bodies	1
Platelet count	Less than 150,000/mm³	1

In reference to Table 1, interpretation was done, as shown in Table 2.

**Table 2:** Interpretation of the hematological score in neonatal sepsis screening. Minimum score = 0; maximum score = 8. The scoring system was adapted from Rodwell et al. (1988) [8] and modified according to our study.

Score	Interpretation
<2	Sepsis less likely
3-4	Sepsis possible
>5	Sepsis very likely

In this study, blood culture was used as the gold standard for diagnosing neonatal sepsis, given its established role in identifying bloodstream infections. However, we acknowledge that pathogen identification, antibiotic susceptibility testing (AST), and contamination rate tracking were not included in the current analysis. This was due to practical limitations, including resource constraints, variability in culture growth, and a focus on evaluating the diagnostic utility of the HSS as a screening tool rather than as a microbiological surveillance study.

Additionally, other biomarkers such as C-reactive protein (CRP) and procalcitonin (PCT), while clinically useful, were not included due to their limited availability during the study period and the variability in cutoff values across institutions. These markers are also costlier, and their inclusion would have introduced bias in a low-resource setting where many neonates are managed without access to these tests.

Statistical analysis

Data were entered into Microsoft Excel (Microsoft Corporation, Redmond, WA) and analyzed using statistical software SPSS version 20 (IBM Corp., Armonk, NY). Descriptive statistics were calculated for baseline characteristics. Categorical variables were compared using the chi-square test.

To determine the independent predictors of culture-positive neonatal sepsis, binary logistic regression analysis was performed. Variables included in the model were hematological parameters (e.g., TLC, total PMN count, I:T ratio, and platelet count) and demographic variables (e.g., gestational age and sex). Results were presented as adjusted odds ratios (AOR) with 95% confidence intervals (CI). A p-value of <0.05 was considered statistically significant.

## Results

This prospective study included a total of 153 neonates. Age distribution of the included neonates is shown in Table 3.

**Table 3:** Distribution of age. Most of the neonates included in the present study were less than seven days old, i.e., 101 (66%).

Age group	Number of cases (%)
<7 days	101 (66%)
7-14 days	34 (22.2%)
14-21 days	10 (6.5%)
21-28 days	8 (5.2%)
Total	153

Based on the clinical scoring system, neonates were classified into groups, namely, sepsis, probable infection, and normal infants, as shown in Table 4.

**Table 4:** Clinical group distribution.

Group	Number of cases (%)
Group 1 (sepsis)	75 (49%)
Group 2 (probable infection)	7 (4.6%)
Group 3 (normal infants)	71 (46.4%)

When the blood culture came back positive, sepsis was diagnosed. When a neonate's blood culture came back negative and they had a significant clinical history or two risk markers for infection, they were categorized as having a likely infection. When there was no clinical history, no risk indicators for infection, and a negative blood culture, newborns were considered normal.

**Figure 1:**
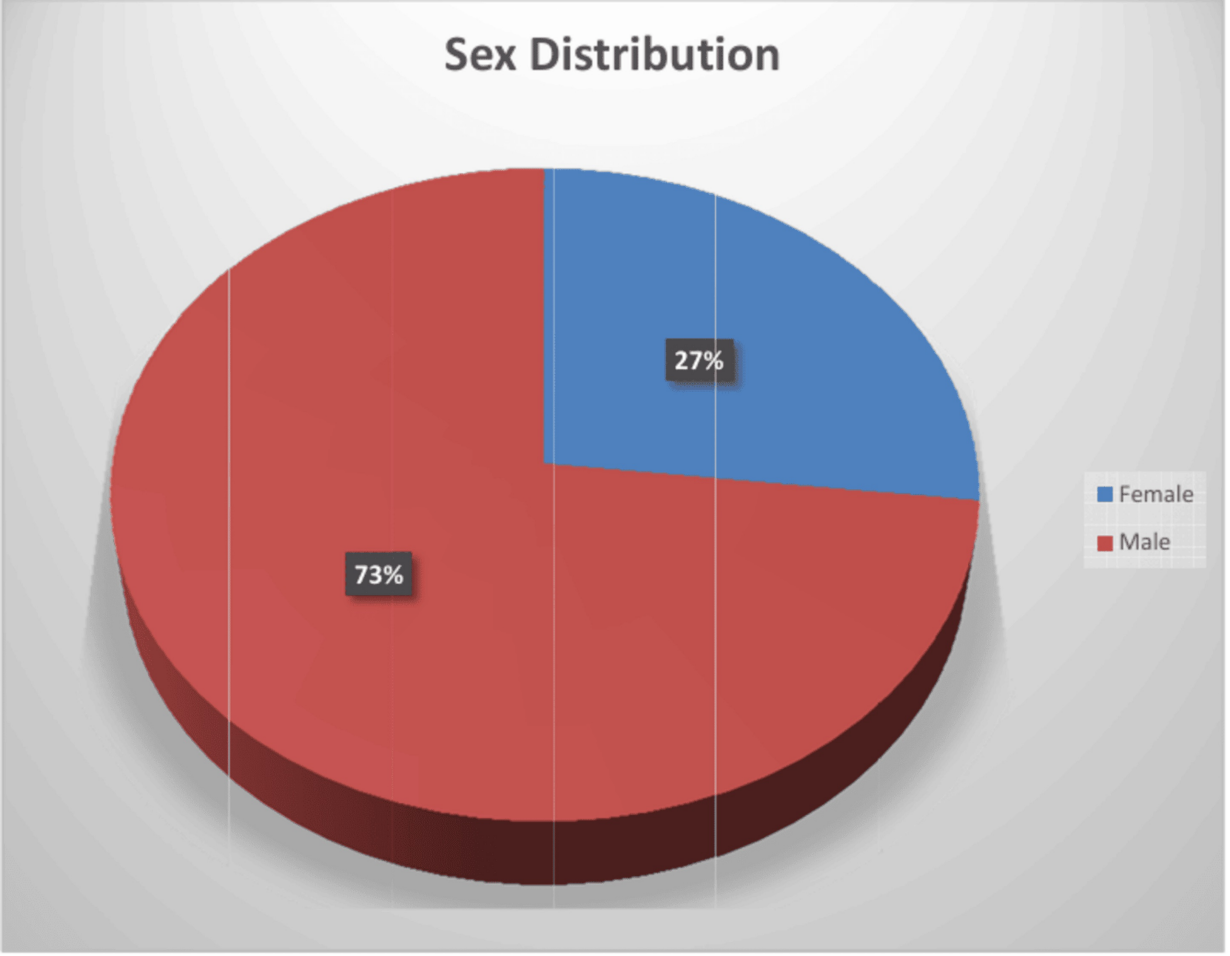
Distribution based on sex.

A total of 112 (73%) of the neonates included in the study were males, and the remaining 41 (26.8%) were females, as shown in Figure 1.

A majority of patients in the present study were term children (133, 86.9%), while the rest were preterm neonates (20, 13.1%). A total of 107 (69.9%) had normal activity after birth, while 46 (30.1%) had poor activity. A total of 131 (85.6%) of neonates had a total WBC count of 5000-21,000, followed by 16 (10.5%) neonates having a total WBC count of <5000, while only six (3.9%) had a total WBC count of more than 21,000.

Of the neonates, 75 (49%) had a PMN count of 1800-5400, followed by 71 (46.4%) neonates who had a PMN count of >5400, while seven (4.6%) had a PMN count of <1800. A majority of neonates (89, 58.2%) had a platelet count of >1.5 lakh, followed by 64 (41.8%) neonates having a platelet count of <1.5 lakh. Of the neonates, 114 (74.5%) had an I:T PMN ratio of <0.12, while 39 (25.5%) had an I:T PMN ratio of >0.12.

The distribution of neonates based on the HSS is shown in Table 5.

**Table 5:** Distribution based on the Hematological Scoring System (HSS). The highest percentage of neonates, 41 (26.8%), had an HSS score of 1, followed by 35 (22.9%) who had a score 2, 20 (13.1%) had a score of 4, 13 (8.5%) had a score of 5, and 11 (7.2%) neonates had a score of 0, while only one (0.7%) had an HSS score of 6.

Score	Frequency	Percentage
0	11	7.2%
1	41	26.8%
2	32	20.9%
3	35	22.9%
4	20	13.1%
5	13	8.5%
6	1	0.7%
Total	153	

The distribution of neonates based on sepsis is shown in Table 6.

**Table 6:** Distribution of neonates based on sepsis.

Sepsis	Number of cases
Possible	55 (35.9%)
Unlikely	81 (52.9%)
Very likely	17 (11.1%)
Total	153

Of the neonates, 78 (51%) had a negative blood culture, while 75 (49%) had a positive blood culture. The association between sepsis and HSS score is described in Table 7.

**Table 7:** Association between sepsis and Hematological Scoring System (HSS) score. The table shows the association between patients’ sepsis status and their HSS score. Chi-square test was applied to know the association (Pearson chi-square value = 293.837, df = 18), and a statistically significant (p < 0.05) association was found between the two, showing the sepsis status of patients varies with their HSS score.

Score	Sepsis			Total	p-value = 0.0001
	Unlikely	Possible	Very likely		
0	11	0	0	11 (7.2%)	
1	41	0	0	41 (26.8%)	
2	29	3	0	32 (20.9%)	
3	0	35	0	35 (22.9%)	
4	0	17	3	20 (13.1%)	
5	0	0	13	13 (8.5%)	
6	0	0	1	1 (0.7%)	
Total	81	55	17	153	

The association between sepsis and blood culture is described in Table 8.

**Table 8:** Association between sepsis and blood culture. The table shows the association between patients’ sepsis status and their blood culture. Chi-square test was applied to know the association (Pearson chi-square value = 60.314, df = 2), and a statistically significant (p < 0.05) association was found between the two, showing the sepsis status of patients varies with their blood culture.

Blood culture	Sepsis			Total	p-value = 0.000001
	Unlikely	Possible	Very likely		
Positive	16	43	16	75 (49%)	
Negative	65	12	1	78 (51%)	
Total	81	55	17	153	

To evaluate the independent contribution of each hematological parameter to the diagnosis of neonatal sepsis, a multivariate logistic regression model was applied. After adjusting for gestational age, sex, and age at admission, the following variables were found to be statistically significant predictors of culture-positive sepsis, as shown in Table 9.

**Table 9:** Logistic regression analysis. This analysis demonstrates that these hematological parameters are independently associated with the likelihood of neonatal sepsis, even after controlling for potential confounders. WBC: white blood cells; PMN: polymorphonuclear neutrophil; I:T PMN ratio: immature-to-total PMN ratio.

Variable	Adjusted odds ratio (AOR)	95% CI	p-value
Abnormal total WBC count	2.4	1.2-4.9	0.012
Abnormal PMN count	3.1	1.5-6.7	0.003
I:T PMN ratio >0.12	2.7	1.3-5.8	0.008
Platelet count <150,000	1.9	1.1-3.2	0.028
Degenerative PMN changes	2.5	1.2-5.2	0.014

## Discussion

In this study, the significance of the HSS in the early diagnosis of late neonatal sepsis was analyzed and interpreted. The study period was from January 2020 to June 2021. A total of 153 clinically suspected cases of neonatal sepsis and/or with risk factors were reported during the study period. The study was conducted at Gandhi Medical College and Associated Hospitals, Bhopal.

The systemic response of neonates to infection is referred to as neonatal sepsis, neonatal septicemia, or sepsis neonatorum. Although laboratory diagnosis necessitates a microbiologic-clinical connection, clinical examination is the primary basis for the early diagnosis of newborn septicemia. A positive blood culture, which takes at least 48 to 72 hours, is necessary for a definitive diagnosis of septicemia. Blood culture remains the gold standard for diagnosis of neonatal sepsis [9]. Out of 153 cases in our study, 73.2% were males and 26.8% were females, which correlates well with Krishnamurthy et al. [10], who reported that sepsis is more common in males.

Our study concluded that there is a greater chance of sepsis noted in patients who have higher hematological scores. With the hematological score of less than 2, sepsis is least likely, as concluded by Narasimha et al. [11], which correlates well with our study. In our study, the total WBC count is a decent predictor of occult bacteremia; however, absolute neutrophil count is more sensitive than total WBC count in predicting occult bacteremia, as studied by Gombos et al. [12], which correlates well with the present study.

Our study shows that neonates are more susceptible to infection because of the immature development of the immune system. To overcome this problem, there are various clinical and hematological parameters suggested to predict neonatal sepsis in advance [13]. In our study, the total platelet count was a good predictor of sepsis, which is in correlation with the study by Narasimha et al. [11].

In our study, the total PMN count had high sensitivity and negative predictive value, and this finding was similar to Makkar et al. [14] and Ramamurthy et al. [15]. The accuracy of sepsis diagnosis is improved by using the HSS. As a result, this can be used as a screening test for sepsis diagnosis. However, it is critical to standardize the method and the interpretation of the results according to a specified protocol.

In addition to the chi-square analysis, we employed logistic regression to adjust for possible confounders such as gestational age and sex. The regression model confirmed that abnormal TLC, elevated I:T ratio, abnormal PMN count, and low platelet count are independent predictors of culture-proven sepsis. These findings are consistent with prior studies by Narasimha et al. [11] and Makkar et al. [14], reinforcing the utility of HSS components as early diagnostic markers.

This multivariate approach strengthens the diagnostic value of HSS and supports its use not just as a preliminary screen but as a tool with real predictive capability in clinical settings.

Automated culture systems, fluorometric detection systems, and DNA probe tests are some of the technologies for the quick detection of bacteria in blood cultures. However, a reliable indicator for predicting newborns with sepsis is the HSS. The prevalence of infection by diverse organisms varies from one institution to the next and even year to year within the same institution, as well as depending on whether the sepsis is early or late.

Limitations

Because the anticoagulant in the vacutainer is constant, a small amount of blood taken may cause the sample to clot or cause hemodilution. Also, morphological changes in leukocytes were observed due to prolonged storage. Our present study consisted of a small study group, most probably due to the COVID constraints, resulting in a lack of statistical power. However, a large cohort study is advisable for proper validation. The cut-off values suggested by our study need proper validation with a large study group to draw a conclusion.

This study did not include microbiological details such as specific pathogen profiles, antimicrobial resistance patterns, or blood culture contamination rates. The decision was made to maintain a focused evaluation of hematological parameters within the constraints of a low-resource NICU setting. Future studies with expanded laboratory support should incorporate CRP, procalcitonin, interleukin levels, and molecular assays for a more comprehensive diagnostic assessment.

## Conclusions

The HSS demonstrated high sensitivity and moderate specificity for the early diagnosis of neonatal sepsis in this study. While this tool does not replace blood culture, it can serve as a cost-effective and rapid screening method, especially in resource-limited settings, to help identify neonates at high risk for sepsis. The use of logistic regression confirms that key hematological parameters within the HSS are independently predictive of neonatal sepsis, validating HSS as a reliable and practical diagnostic tool in resource-limited settings.

Although this study did not assess clinical outcomes such as mortality or hospital length of stay, the timely identification of sepsis through HSS may support earlier clinical decision-making and antibiotic initiation, which could potentially contribute to improved outcomes. Further prospective studies are warranted to explore the impact of HSS-guided interventions on mortality, morbidity, and healthcare utilization in neonatal care.
